# Erythema Annulare Centrifugum: A Rare Skin Manifestation of Hashimoto Thyroiditis

**DOI:** 10.7759/cureus.9906

**Published:** 2020-08-20

**Authors:** Palwasha Jalil, Sadia Masood, Saira Fatima

**Affiliations:** 1 Dermatology, Aga Khan University Hospital, Karachi, PAK; 2 Histopathology, The Aga Khan University, Karachi, PAK

**Keywords:** annular erythema, hashimoto thyroiditis, indurated plaques

## Abstract

Erythema annulare centrifugum (EAC) is an unusual skin condition appearing as recurrent erythematous annular eruptions associated with autoimmune disorders, infections, and various neoplastic conditions. We present a very rarely reported association of EAC with Hashimoto thyroiditis (HT) in a young male. A 26-year-old male recently diagnosed as case of HT presented in the dermatology clinic with nine-month history of non-itchy persistent annular lesions on the body. The morphology and biopsy of lesions confirmed the diagnosis of EAC.

HT is a part of the spectrum of autoimmune thyroid diseases with its own specific cutaneous manifestations. Our case also depicts the impact of antigen-antibody related immunological reaction, which might be involved in the development of both HT and EAC, and it could be the stages of the same pathological condition of two different clinical presentations.

## Introduction

Erythema annulare centrifugum (EAC) is an unusual skin condition that appears as recurrent erythematous eruptions in the form of small and large annular plaques [1]. It is associated with various autoimmune disorders, infections, and few neoplastic conditions. However, in most of the cases, the exact cause is not clearly identified. The disease is classified into deep and superficial types, but these types are not specifically associated with any systemic condition [2].

The term erythema annulare centrifugum was first described by Darier in 1916 [3]. Along with usually described associations of EAC, literature has reported various unusual associations of EAC, such as chronic active hepatitis, polyglandular syndrome, alopecia areata, and vitiligo [4].

Hashimoto thyroiditis (HT) is part of the spectrum of autoimmune thyroid diseases with its own specific cutaneous manifestations [5]. In HT, thyroid cells are destroyed by the various antibody-mediated immune processes. It is highly associated with other autoimmune diseases, including type 1 diabetes mellitus, coeliac disease, adrenal insufficiency, and pernicious anemia [6]. We report an interesting case of EAC associated with HT that presented as lesions of figurate erythema over the body. HT is associated with various skin conditions, but the association with EAC is very rarely reported previously.

## Case presentation

**Figure 1:**
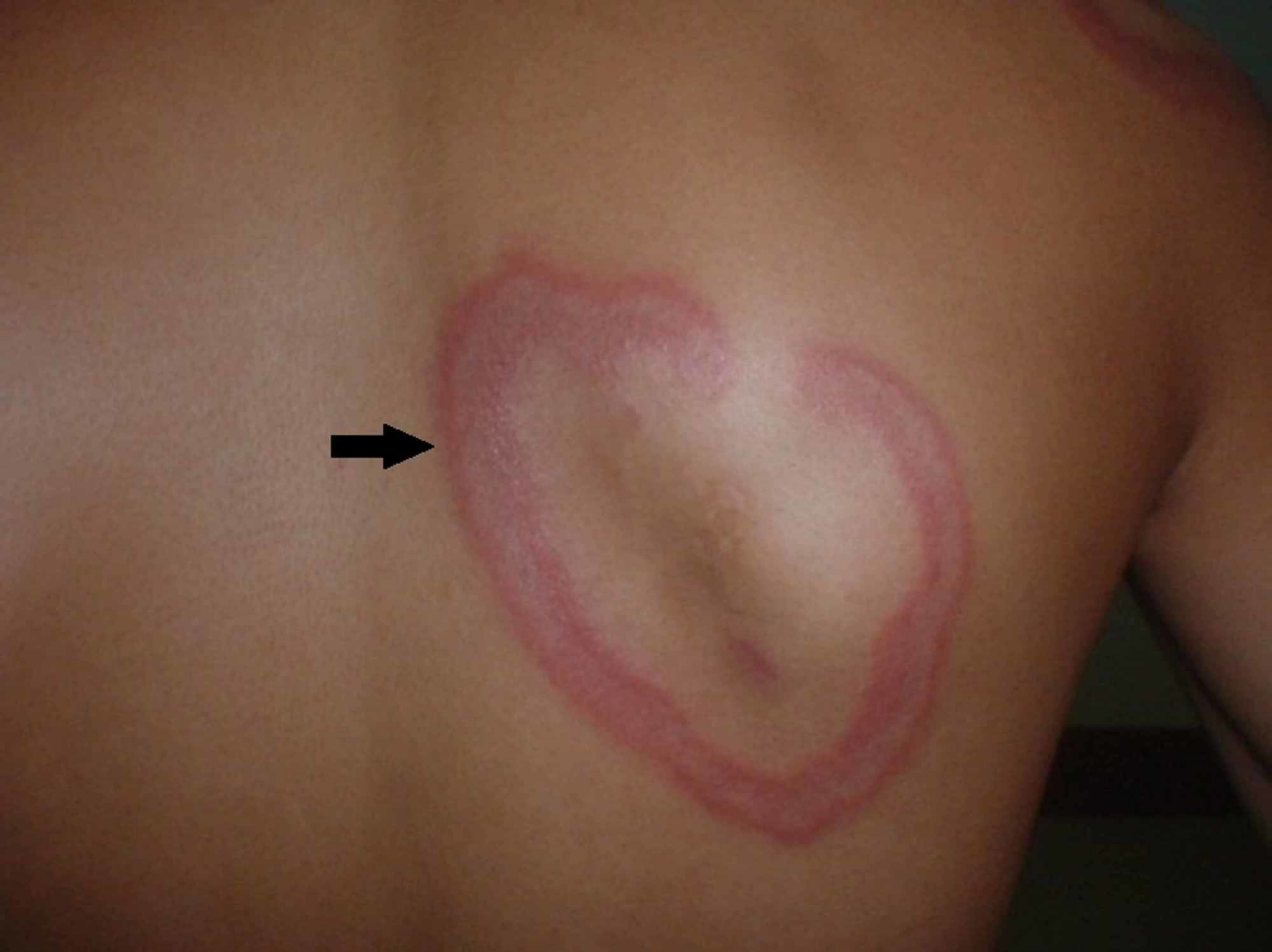
Annular erythematous plaque with central clearance

A 26-year-old male was referred to skin clinic with nine-month history of non-itchy persistent rashes over the body. They initially begin as small, raised papules and enlarged by peripheral extension to form annular lesions. After a variable time period of weeks and months, the individual lesions used to disappear, often to be replaced by new ones. It first appeared on his nose as small pink raised papule, which slowly enlarged and formed rings and gradually spread to his trunk and extremities. The lesions were asymptomatic, but they were cosmetically disturbing to the patient. He was recently diagnosed as a case of HT and referred from endocrinology clinic for his skin rash. There was no history of fever, weight loss, and insect bite, traveling or taking any new drug except oral thyroxin. Local examination revealed annular erythematous papules having raised infiltrated edges with central flattening and the fading of erythema (Figure 1).

**Figure 2:**
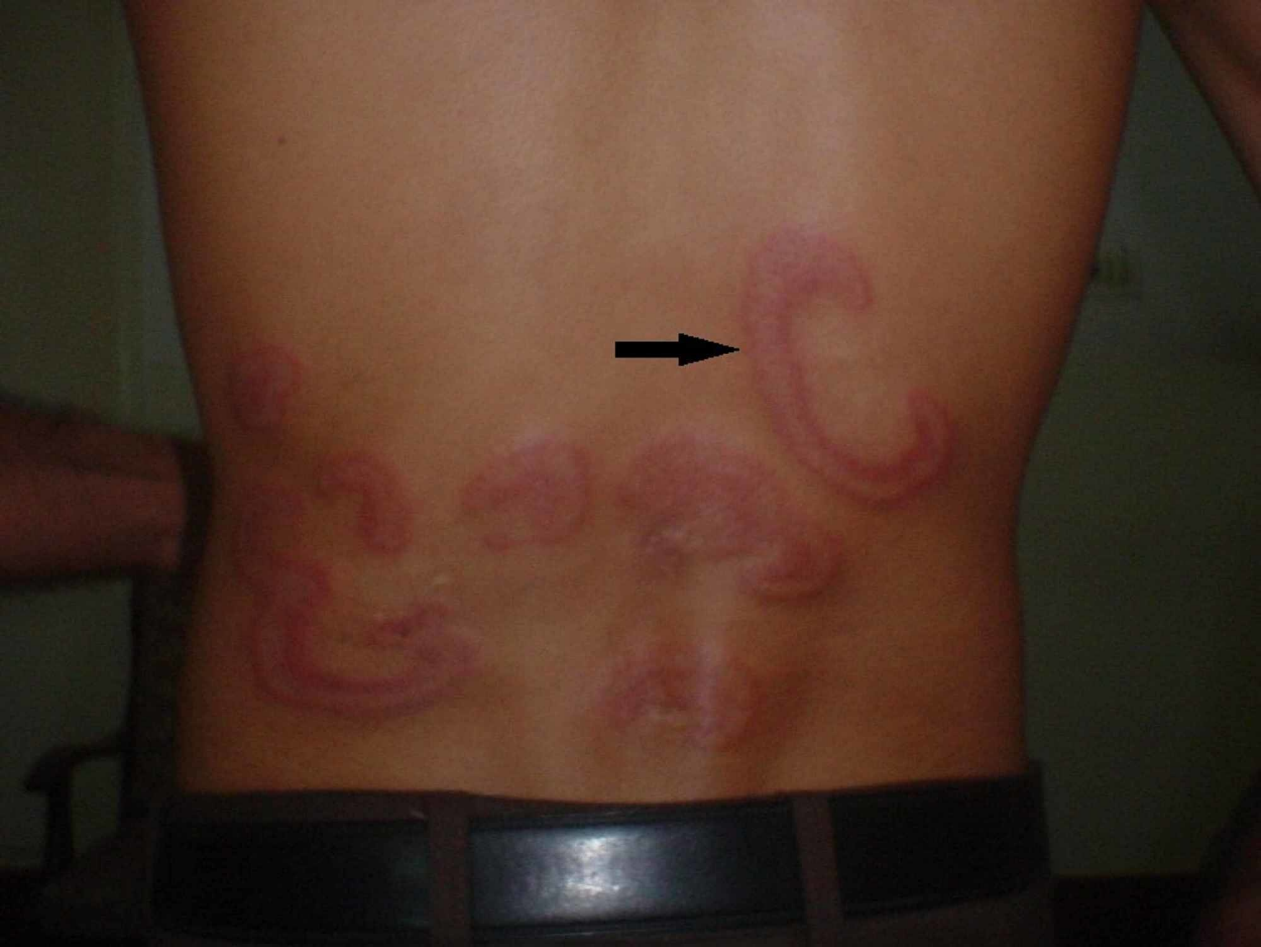
Figurate erythema with well-defined indurated borders on the back

**Figure 3:**
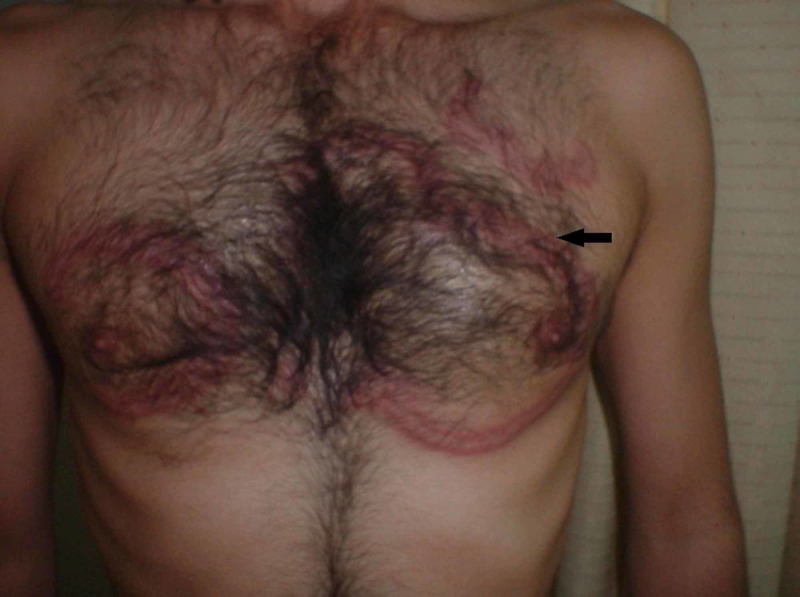
Figurate erythema with well-defined indurated borders on the chest

The margins were indurated, varying in width from 6 to 8 mm. Some lesions were extending irregularly to leave arciform segments. They were present on the chest, back, buttocks and legs with sparing of palms and soles. The polycyclic lesions varied from 10 to 20 cm (Figures 2, 3).

**Figure 4:**
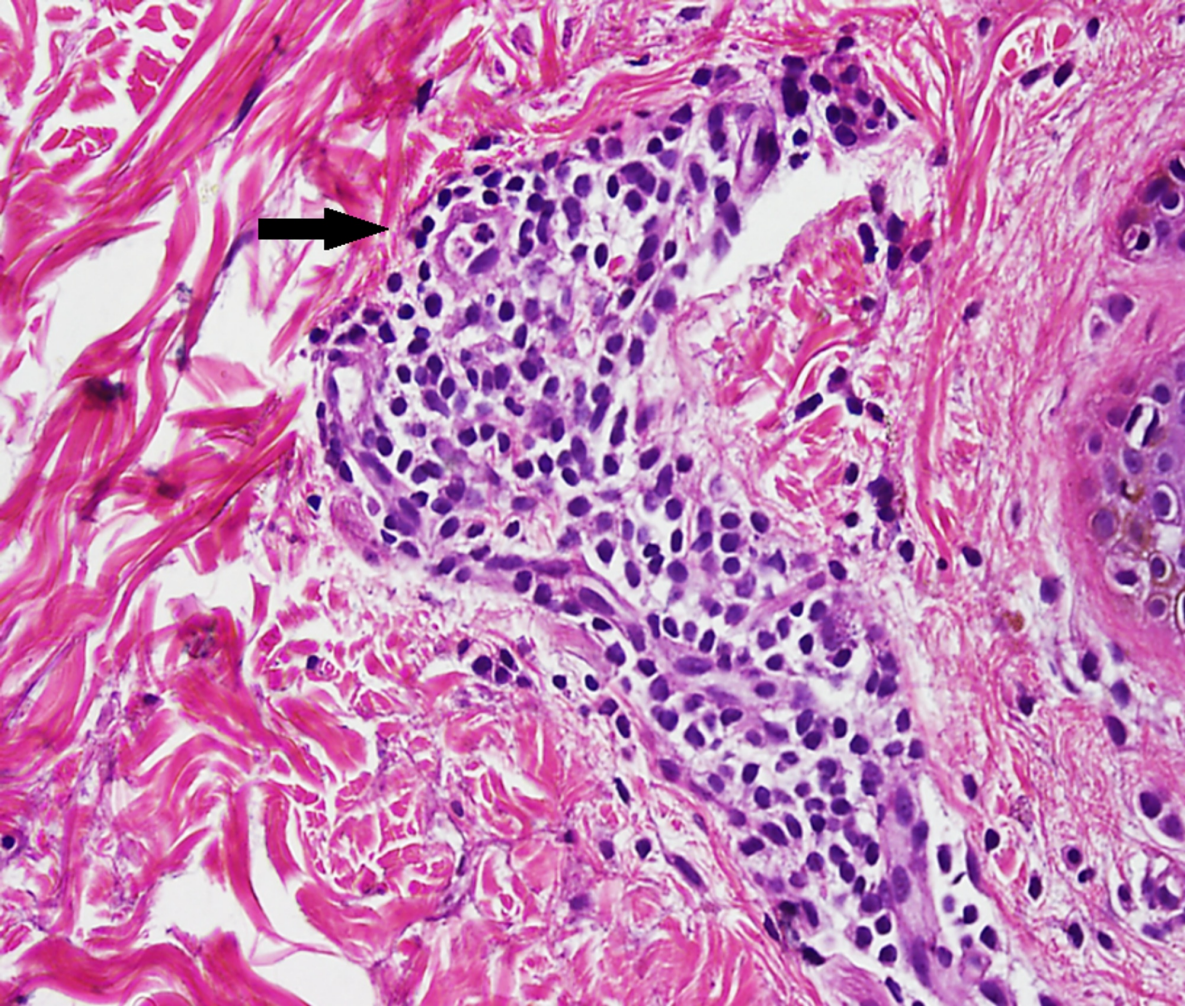
Perivascular lymphocytes in coat sleeve pattern

**Figure 5:**
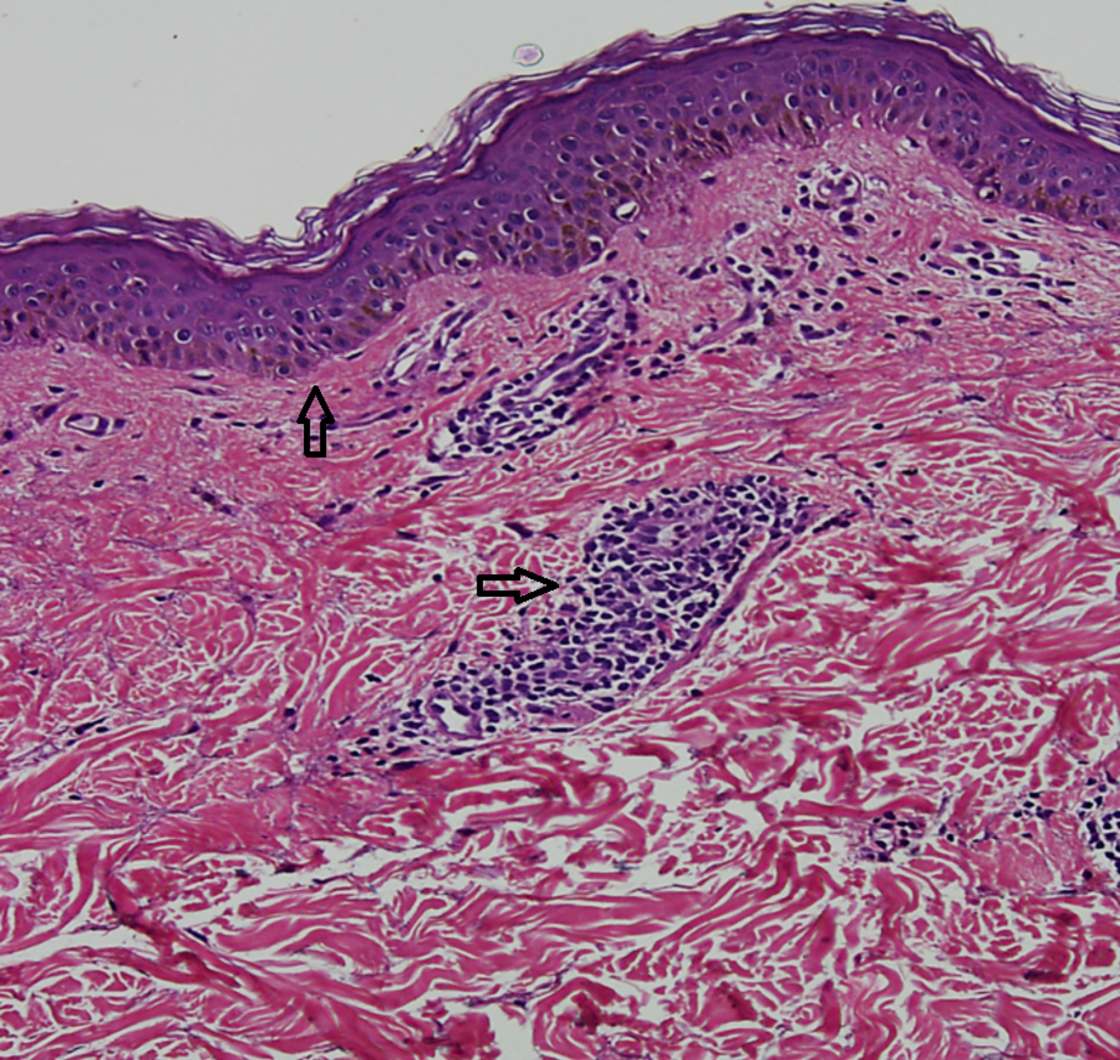
Mild hyperkeratosis, flattened rete pegs. Dermis exhibits tight perivascular inflammatory infiltrate

The skin was generally dry. Systemic review revealed no abnormality. Hemogram, liver function tests, Venereal Disease Research Laboratory test (VDRL), kidney function tests, and chest x ray were normal. Potassium hydroxide (KOH) skin preparation was done to exclude the presence of fungal hyphae. The diagnosis was then confirmed by the skin biopsy, which revealed ‘sleeve-like’ lymphohistiocytic infiltrate in the middle and lower dermis (Figures 4, 5).

Topical steroids were used for the treatment of inflammatory lesions, and he was advised to come for follow-ups to monitor the progress of disease.

## Discussion

The descriptive term erythema annulare centrifugum comprises of redness (erythema), ring form appearance (annulare), and spread from center (centrifugum) [7]. The age of occurrence for EAC is variable, and it has been reported from infancy to ninth decade of life [1].

EAC is actually a skin hypersensitivity reaction to various underlying disorders like several type of fungal, bacterial, and viral infections, different malignancies, and various drugs like lactone, gold, cimetidine, lactone, antimalarials, and amitriptyline [8,9]. It is also reported to be associated with some disorders like Graves’ disease, sarcoidosis, Crohn's disease, chronic active hepatitis, and very rarely with hypothyroidism [10,11]. It is usually a self-limiting condition and resolves with treatment of underlying disease. Hence, a search for the underlying disorder and its treatment is the primary therapy [7].

HT is an autoimmune disease, and the initiating process is still not well understood. Antibodies usually bind thyroid-stimulating hormone receptors, and thyrotrophic receptor blocking antibodies may also contribute to impairment in thyroid function that results in inadequate thyroid hormone production and secretion [5,6]. It is most frequently associated with other autoimmune conditions like type 1 diabetes mellitus, coeliac disease, pernicious anemia, and adrenal insufficiency. Cutaneous manifestations of HT are decrease sweating, dryness of skin, and diffuse hair loss [12].

The current case report highlights an interesting clinical association of HT with the unusual skin lesions of EAC. In our patient, cutaneous findings appeared three months before the clinical diagnosis of HT. The EAC is mainly characterized by superficial and deep type of lesions. The deep type is clinically presented as an annular non-itchy and non-scaly indurated plaque [8], where histopathology shows a dense perivascular infiltrate in the middle and lower dermis [13], and the superficial form clinically presents as well-demarcated ring-like scaly plaques [1-4]. Our patient had the deep type of EAC having a characteristic feature of non-scaly well-demarcated indurated plaques.

As discussed above, the EAC is associated with different disorders and considered as a delayed type of hypersensitivity reaction that is usually triggered by different antigens [12]. However, the exact etiology for its appearance in autoimmune diseases is still unclear. One hypothesis is that the T lymphocytes recognize antigen-presenting cells on surface receptors of thyroid cells by autologous HLA molecules [14]. An interaction between the HLA molecule and antigenic peptide is necessary. The literature supported this hypothesis by the appearance of parallel course of autoimmune diseases and annular skin lesions in many cases [15]. However, there is no published data where the autoimmune mechanism and association have been clearly identified. Various genetic factors usually influence the effect of helper T-cell recognition and autoantigen presentation [16]. The current case also depicts the impact of antigen-antibody related immunological reaction, which might be involved in the development of both HT and EAC, and it could be the stages of a same pathological condition of two different clinical presentations [1].

The rare association of EAC with HT needs to be reported as the underlying immunologic process of primary disease can affect any system, including the skin. Therefore, the disease activity of HT would show rapid progression of skin problem that can also have rapid improvement with treatment of underlying disease.

## Conclusions

EAC is rarely associated with HT. Familiarity with this association would prompt physicians to properly examine and then investigate for HT if required. EAC is a very rare condition, the documentation of cases and information are sparse, and the current observations showed that EAC could actually be a set of many unclassified skin lesions.
